# Computer vision syndrome among computer office workers in a developing country: an evaluation of prevalence and risk factors

**DOI:** 10.1186/s13104-016-1962-1

**Published:** 2016-03-09

**Authors:** P. Ranasinghe, W. S. Wathurapatha, Y. S. Perera, D. A. Lamabadusuriya, S. Kulatunga, N. Jayawardana, P. Katulanda

**Affiliations:** Department of Pharmacology, Faculty of Medicine, University of Colombo, Colombo, Sri Lanka; Ministry of Health Care and Nutrition, Colombo, Sri Lanka; Department of Clinical Medicine, Faculty of Medicine, University of Colombo, Colombo, Sri Lanka

**Keywords:** Computer vision syndrome, Prevalence, Risk factors, Sri Lanka

## Abstract

**Background:**

Computer vision syndrome (CVS) is a group of visual symptoms experienced in relation to the use of computers. Nearly 60 million people suffer from CVS globally, resulting in reduced productivity at work and reduced quality of life of the computer worker. The present study aims to describe the prevalence of CVS and its associated factors among a nationally-representative sample of Sri Lankan computer workers.

**Methods:**

Two thousand five hundred computer office workers were invited for the study from all nine provinces of Sri Lanka between May and December 2009. A self-administered questionnaire was used to collect socio-demographic data, symptoms of CVS and its associated factors. A binary logistic regression analysis was performed in all patients with ‘presence of CVS’ as the dichotomous dependent variable and age, gender, duration of occupation, daily computer usage, pre-existing eye disease, not using a visual display terminal (VDT) filter, adjusting brightness of screen, use of contact lenses, angle of gaze and ergonomic practices knowledge as the continuous/dichotomous independent variables. A similar binary logistic regression analysis was performed in all patients with ‘severity of CVS’ as the dichotomous dependent variable and other continuous/dichotomous independent variables.

**Results:**

Sample size was 2210 (response rate—88.4 %). Mean age was 30.8 ± 8.1 years and 50.8 % of the sample were males. The 1-year prevalence of CVS in the study population was 67.4 %. Female gender (OR: 1.28), duration of occupation (OR: 1.07), daily computer usage (1.10), pre-existing eye disease (OR: 4.49), not using a VDT filter (OR: 1.02), use of contact lenses (OR: 3.21) and ergonomics practices knowledge (OR: 1.24) all were associated with significantly presence of CVS. The duration of occupation (OR: 1.04) and presence of pre-existing eye disease (OR: 1.54) were significantly associated with the presence of ‘severe CVS’.

**Conclusions:**

Sri Lankan computer workers had a high prevalence of CVS. Female gender, longer duration of occupation, higher daily computer usage, pre-existing eye disease, not using a VDT filter, use of contact lenses and higher ergonomics practices knowledge all were associated with significantly with the presence of CVS. The factors associated with the severity of CVS were the duration of occupation and presence of pre-existing eye disease.

## Background

“Computer Vision Syndrome” (CVS), is defined by the American Optometric Association as a complex of eye and vision problems related to the activities which stress the near vision and which are experienced in relation to or during the use of computers [1]. It encompasses a group of visual symptoms which crop up from the extended viewing of the video display terminal (VDT), when the demands of the task exceed the abilities of the viewer. Symptoms of CVS includes; dry and irritated eyes, eye strain/fatigue, blurred vision, red eyes, burning eyes, excessive tearing, double vision, headache, light/glare sensitivity, slowness in changing focus and changes in colour perception [2]. It is estimated that nearly 60 million people suffer from CVS globally, and that a million new cases occur each year [3]. In the twenty first century personal computers are one of the commonest office tools, used in almost all institutions/organizations, for a wide variety of vocational and/or non-vocational purposes. Hence, it is likely that CVS will continue to create a significant and growing contribution to reduced productivity at work, whilst also reducing the quality of life of the computer office worker.

Estimates of the prevalence of eye problems associated with VDTs vary enormously, depending on the sample tested, research methods employed and study instrument used [4, 5]. In a review on CVS, Thomson indicated that up to 90 % of computer users may experience symptoms related to CVS after prolonged computer usage [4]. Other studies estimate that the prevalence of CVS ranges from 75 to 90 % among computer users [6]. A lower prevalence of asthenopia (eye strain/fatigue) among computer users has been observed in Italy (n = 212; 31.9 %), India (n = 400; 46.3 %), Australia (n = 1000; 63.4 %) and Spain (n = 35; 68.5 %) [7–10]. However, most of the studies on CVS prevalence have been among a limited number of computer workers and usually conducted within a single institution/organization.

South Asia, commonly known as the Indian sub-continent, is home to almost one-quarter of the world’s population and is comprised of many diverse ethnic, linguistic and religious groups. India, Pakistan, Bangladesh, Nepal, Sri Lanka, Bhutan and Maldives are the countries of the region. The region has undergone rapid socio-economic development and technological advancement during the last few decades, with resultant increase in computer literacy and usage. Hence, it is likely that South Asian computer office workers also have a significant prevalence of CVS, with its associated loss of productivity and compromised quality of life. Similar to other countries, the few South Asian studies on CVS prevalence that are currently available in the literature are small scale and based in a single institution. At present there are no large scale nationally representative studies on the prevalence of CVS among computer office workers from the South Asian region or from the rest of the world. The present study aims to describe the prevalence of CVS and its associated factors among a nationally representative sample of Sri Lankan computer office workers.

## Methods

### Study population and sampling

Detailed sampling has been described elsewhere; a brief summary is presented here [11]. The study locations were two telecommunication institutes and a computer training institute with branches in all of the nine provinces of Sri Lanka [9]. Two thousand five hundred computer office workers were invited for the study. The number of participants to be invited from each province was determined by the probability proportionate to sample size (PPS) method depending on population data for each province as determined by the Department of Census and Statistics, Sri Lanka [12]. Inclusion criteria was computer workers who used computers to complete their job tasks for at least 2 h per day, and had worked in the current position for at least 12 months. A list of employees satisfying the inclusion criteria was obtained from the human resources department of the respective institutes. Simple random sampling by using computer generated random numbers was used to randomize this final list of employees and the selected computer workers were invited for the study. Informed written consent was obtained from each study participant. Ethical approval for the study was obtained from the Ethics Review Committee of the Faculty of Medicine, University of Colombo, Sri Lanka and the study was conducted between May and December 2009.

### Study instruments

A self administered questionnaire was used to collect socio-demographic data, symptoms of CVS, details of computer usage, potential risk factors, evaluate current workstations and knowledge on ergonomics and ergonomic practices. Questions on symptoms of CVS were adapted from a previous study done by Gangamma et al. [2]. Presence of pain in and around the eyes, headache, blurred near vision, blurred distant vision, dry eyes, sore/irritated eyes, red eyes, excessive tearing, double vision, twitching of eye lids and changes in visualizing colours were assessed as symptoms of CVS. The participants were asked about the presence of the above symptoms during the previous year. To be considered as a symptom of CVS the symptoms had to last for at least 1 week during the previous year. Presence of any one of the above symptoms, either intermittently or continuously for at least 1 week during the previous year was considered as ‘presence of CVS’. In the absence of a uniform diagnostic criterion, we adopted the above criteria after perusal of previous research and by obtaining expert opinions [2, 5, 13, 14]. Age, gender and province of residence were assessed as demographic data. The information collected on computer usage were duration of occupation, daily computer usage measured in number of hours, duration of continuously staring at monitor and purpose for which the computer is frequently used. Questions on potential risk factors were prepared after reviewing the articles on CVS in the literature. Any pre-existing eye disease, use of contact lenses, taking a regular rest during computer work, type and size of current monitor, current use of a VDT filter, current distance between face and monitor, current vertical height from center of screen to gaze line, adjustment of screen brightness to suit the surrounding and current room environment (positioning of room lights, window curtains) were the risk factors studied. Individual workstations were evaluated by the investigators using the validated Occupational Safety and Health Administration (OSHA) VDT workstation checklist [15]. Set of expert-validated self-administered questions were used to evaluate the participant knowledge and awareness of ergonomics, and the extent to which the principles of ergonomics were put into practice in the work-place. Ten pictorial questions evaluated participants’ knowledge on correct postures and equipment placement, each correct answer was given one mark (total score-10).

### Statistical methods

Data was analyzed using Statistical Package for Social Sciences (SPSS) software, version 14. Descriptive data was presented as percentages or as mean ± standard deviations. The dependant variable in this study was presence of CVS. Significance of associations was tested using Chi square for categorical variables and Student’s *t* test for continuous variables.

**Fig. 1:**
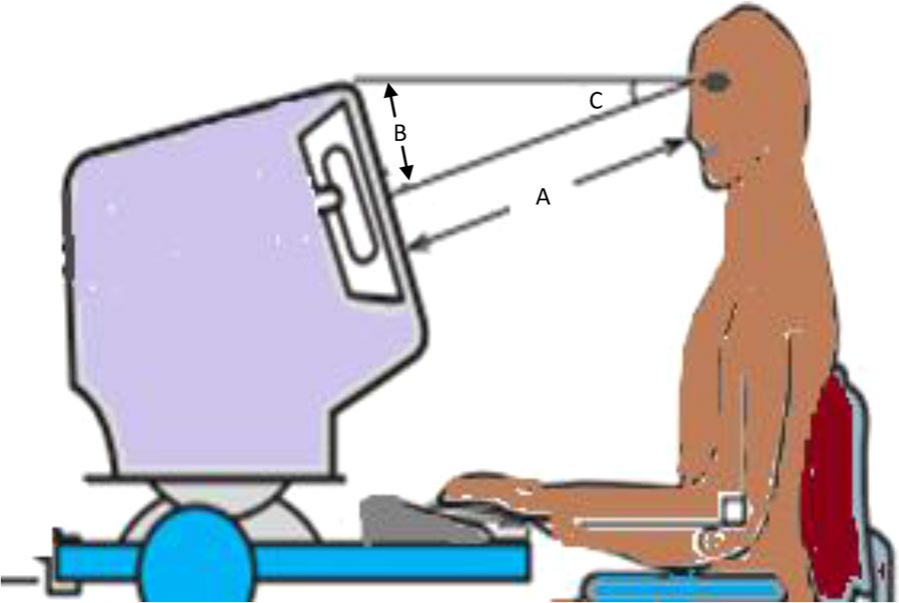
Angle of gaze

Angle of gaze to the computer monitor (C) (Fig. 1) was calculated using the two measurements; distance between face and the monitor (A) and the distance between straight ahead gaze line and center of the screen (B). The formula used to calculate the angle of gaze to the computer monitor was C = tan(−1) (B/A) [16]. Subjects were divided into two groups based on the presence or absence of CVS. A binary logistic regression analysis was performed in all patients with ‘presence of CVS’ as the dichotomous dependent variable (0 = CVS absent; 1 = CVS present) and age, gender (0 = male; 1 = female), duration of occupation (years), daily computer usage (hours), pre-existing eye disease (0 = absent; 1 = present), not using a VDT filter (0 = no; 1 = yes), adjusting brightness of screen (0 = no; 1 = yes), use of contact lenses (0 = no; 1 = yes), angle of gaze and ergonomic practices knowledge as the continuous/dichotomous independent variables. The explanatory independent variables that were associated with the dependent variable in univariate analysis (p < 0.25) were selected to be included in the regression analysis. The explanatory variables selected above were subsequently included in a binary logistic regression model, a backward elimination procedure was used and a p value of 0.10 was considered as the cut-off for removal of variables. A similar binary logistic regression analysis with above dependant and independent variables was also performed separately for both males and females.

Participants with CVS were classified into two sub-groups depending on severity of CVS: (1) mild to moderate cases: subjects having seven or less symptoms with all the symptoms disappearing after a short rest; (2) severe cases: subjects reported more than 7 symptoms and/or subjects having at least one symptom that does not disappear even after a short rest. These criteria were defined according to expert opinion, due to lack of previously defined classifications. A binary logistic regression analysis was performed in all patients with ‘severity of CVS’ as the dichotomous dependent variable (0 = mild-moderate CVS; 1 = severe-CVS) and duration of occupation (years), daily computer usage (hours), angle of gaze, pre-existing eye disease (0 = absent; 1 = present), using a VDT filter (0 = no; 1 = yes), adjusting brightness of screen (0 = no; 1 = yes) and ergonomic practices knowledge as the continuous/dichotomous independent variables. The explanatory independent variables that were selected using a similar method as described before and a backward elimination procedure was used and a p value of 0.10 was considered as the cut-off for removal of variables. In all analyses a p value ≤0.05 was considered statistically significant.

## Results

### Socio-demographic characteristics

Sample size was 2210 (response rate—88.4 %). Mean age was 30.8 ± 8.1 years (range 18–60 years) and 50.8 % of the sample were males. A majority (48.1 %) of the study population belonged to the age category 20–29 years with 46.5 % males and 49.6 % females being in this age group. Seventy five percent of the study population had worked between 1 and 5 years in their current position. Of the male participants, 45.6 % worked 6–9 h per day with a computer, compared to 42.8 % of the female participants and 44.3 % of the entire study population. Pre-existing eye diseases, which included presence of cataract, glaucoma, presbyopia, myopia and oculomotor abnormalities, were present in 25 % (n = 552). Sample characteristics are summarized in Table 1.

**Table 1:** Characteristics of the study population

	All	Males	Females
Province (number required^a^)			
Western (1100)	1116 (50.5 %)	548 (48.8 %)	568 (52.2 %)
Central (240)	280 (12.7 %)	92 (8.2 %)	188 (17.3 %)
Sabaragamuwa (140)	170 (7.7 %)	90 (8.1 %)	80 (7.4 %)
North-Western (160)	164 (7.4 %)	80 (7.1 %)	84 (7.7 %)
Southern (160)	162 (7.3 %)	82 (7.3 %)	80 (7.4 %)
Eastern (80)	84 (3.8 %)	76 (6.8 %)	8 (0.7 %)
Northern (70)	84 (3.8 %)	76 (6.8 %)	8 (0.7 %)
North-Central (80)	82 (3.7 %)	42 (3.7 %)	40 (3.7 %)
Uva (60)	68 (3.1 %)	36 (3.2 %)	32 (2.9 %)
Age			
<20 years	100 (4.5 %)	26 (2.3 %)	74 (6.8 %)
20–29 years	1062 (48.0 %)	522 (46.5 %)	540 (49.6 %)
30–39 years	740 (33.5 %)	400 (35.7 %)	340 (31.3 %)
≥40 years	308 (13.9 %)	174 (15.5 %)	134 (12.3 %)
Number of working years in current position			
1–5 years	1670 (75.6 %)	846 (75.4 %)	824 (75.7 %)
6–10 years	210 (9.5 %)	116 (10.3 %)	94 (8.6 %)
11–15 years	192 (8.7 %)	84 (7.5 %)	108 (10.0 %)
15 years and more	138 (6.2 %)	76 (6.8 %)	62 (5.7 %)
Number of working hours with computer/day			
2–5 h	532 (24.1 %)	230 (20.5 %)	302 (27.8 %)
6–9 h	978 (44.2 %)	512 (45.6 %)	466 (42.8 %)
>9 h	700 (31.7 %)	380 (33.9 %)	320 (29.4 %)

^a^Number required from each province, based on percentage computer usage in each province and total provincial population (PPS method)

### Prevalence of CVS

The 1-year prevalence of CVS in the study population was 67.4 %. Prevalence of CVS was significantly greater in females (69.5 %) than in males (65.4 %) (p < 0.05). The most commonly reported complaint was headache (45.7 %), followed by dry eyes (31.1 %), whereas the least common complaint was changes in visualizing colours (9.3 %). The prevalence of each symptom in all participants, males and females are presented in Table 2. The prevalence of headache was significantly higher in females, while red eyes, changes in visualizing colours and excessive tearing were more prevalent in males (Table 2). There was no significant difference between the genders for any of the other symptoms of CVS (Table 2).

**Table 2:** One year prevalence of CVS symptoms lasting for at least one week during the previous year and severity

	N	All prevalence % (95 % CI) (n = 2210)	Males prevalence % (95 % CI) (n = 1122)	Females prevalence % (95 % CI) (n = 1088)	p value*
Pain in and around the eyes	634	28.7 (26.8–30.6)	27.8 (25.2–30.5)	29.6 (26.9–32.4)	NS
Headache	1010	45.7 (43.6–47.8)	38.1 (35.2–41.0)	53.5 (50.5–56.5)	<0.05
Blurred near vision	526	23.8 (22.0–25.6)	24.6 (22.1–27.2)	23 (20.5–25.6)	NS
Blurred distant vision	444	20.1 (18.4–21.8)	19.6 (0.4–40.5)	20.6 (18.2–23.1)	NS
Dry eyes	688	31.1 (29.1–33.1)	30.8 (28.1–33.6)	31.4 (28.7–34.3)	NS
Sore/irritated eyes	578	26.2 (24.4–28.1)	25.3 (22.8–27.9)	27 (24.4–29.8)	NS
Red eyes	400	18.1 (16.5–19.8)	21.9 (19.5–24.5)	14.2 (12.1–16.4)	<0.05
Excessive tearing	460	20.8 (19.1–22.6)	23.2 (20.7–25.8)	18.4 (16.1–20.8)	<0.05
Double vision	302	13.7 (12.3–15.2)	15 (12.9–17.2)	12.3 (10.4–14.4)	NS
Twitching of eye lids	452	20.5 (18.8–22.2)	21 (18.7–23.5)	19.9 (17.6–22.5)	NS
Changes in visualizing colours	206	9.3 (8.1–10.6)	12.7 (10.8–14.7)	5.9 (4.6–7.5)	<0.05
Severity of CVS					
Mild-moderate cases	300	42.1 (39.1–45.3)	44.7 (40.4–49.5)	55.3 (49.7–60.9)	<0.05
Severe cases	412	57.9 (54.7–60.9)	54.9 (50.0–59.6)	45.1 (39.1–50.3)	NS

*CVS* computer vision syndrome, *NS* not significant, *N* number of subjects with complaints

* p value for males vs. females

The province of residence was significantly associated with prevalence of CVS (p < 0.001). Prevalence of CVS was highest in the Western province (74.0 %) and lowest in Southern province (39.5 %). Prevalence of CVS significantly increased with increasing age of the computer user (p < 0.01). Prevalence was highest (72.7 %) among those aged 40 years or more and lowest (58.0 %) among those aged less than 20 years. The mean age of those with CVS (31.2 ± 7.8 years) was significantly higher than the mean age of those without CVS (29.9 ± 8.6 years) (p < 0.01). This relationship was independently observed in females but not in males. Majority of subjects with CVS had severe symptoms (57.9 %), while 42.1 % had mild to moderately severe symptoms (p < 0.05). The prevalence of mild-moderate and severe symptoms in all participants, males and females are presented in Table 2. Mild-moderate symptoms were significantly more prevalent in females (Table 2). In study participants with CVS, 13.2 % (n = 196) used lubricating eye drops and 27.9 % (n = 416) used computer glasses.

### Workstation evaluation

Workstations were evaluated by using the OSHA VDT workstation checklist. Among 2210 workstations evaluated, a significant majority of the workstations (88.4 %, n = 1954) were non-compliant with the OSHA VDT workstation checklist. In those with non-compliant workstations (n = 1954) prevalence of CVS was 68.1 % whereas prevalence of CVS was lower among those with OSHA compliant workstations (62.5 %) (p < 0.05). In addition among 1490 study participants who suffered from CVS, 89.3 % had non-compliant workstations.

### Factors associated with CVS and severity

Prevalence of CVS among subjects with pre-existing eye disease (87.3 %) was significantly higher than the prevalence of CVS among those without a pre-existing eye disease (60.8 %) (p < 0.001). Prevalence of CVS was significantly higher in contact lenses users (93.1 %) than in others (66.7 %) (p < 0.001). Mean duration of occupation was significantly higher in patients with CVS (5.1 ± 5.7 years) than that in patients without CVS (3.6 ± 4.8 years) (p < 0.05). Mean daily computer usage in those with and without CVS is 7.8 ± 3.3 h and 6.7 ± 3.5 h respectively (p < 0.05). Duration of continuously staring at the monitor was also significantly associated with the prevalence of CVS (p < 0.05). Participants with CVS continuously stare at the monitor for a mean duration of 36.8 ± 31.7 min whereas those without CVS continuously stare at the monitor only for a mean duration of 29.8 ± 26.8 min. Prevalence of CVS in those using a monitor without a filter (69.6 %) is significantly higher when compared to those using a monitor with a filter (63.0 %) (p < 0.05). In contrast to this, type of the monitor (CRT—69 % or LCD—69.2 %) was not significantly associated with the prevalence of CVS. Another significant factor was whether the computer user adjusts the screen brightness/contrast to suit the surrounding (p < 0.001). Prevalence of CVS in those who adjust the screen brightness/contrast to suit the surrounding and those who does not was 63.9 % and 72.1 % respectively. Mean angle of gaze to the monitor was significantly higher in those with CVS (31.9° ± 14.5°) than in those without CVS (29.9° ± 14.8°) (p < 0.01). Factors such as monitor size, distance between face and the monitor, taking breaks, positioning of lights and curtain covered windows were not associated with the prevalence of CVS or its severity.

Those with severe CVS had a longer duration of occupation (5.5 ± 5.9 years) than those with mild-moderate CVS (4.4 ± 5.5 years) (p < 0.001). The ergonomics practices knowledge of those with mild-moderate CVS was higher (score-6.6 ± 1.6) than in those with severe CVS (score-5.9 ± 2.0) (p < 0.05). A significant majority of those with pre-existing eye diseases (65.3 %) had severe disease. The angle of gaze was significantly higher in those with Severe CVS (33.4 ± 14.2) than in those with mild-moderate CVS (30.8 ± 15.5) (p < 0.05). Age, duration of daily computer use, time spent continuously staring at the monitor without a break, adjustment of brightness of monitor to suit surrounding environment and usage of a VDT filter was not associated with the severity of CVS.

### Knowledge and awareness of ergonomics

Majority of study participants were not aware about the term ‘Ergonomics’ (70.1 %), while only 14.0 % defined the term correctly. In those who have ever heard of the term ‘Ergonomics’, 39.1 % had heard of it at the present workplace/institute, 32.5 % via the internet, 16.6 % via media (television/radio/newspapers), 14.6 % at a workshop or conference, 14.2 % from colleagues and 3.6 % from a health care professional. In those who had heard of the term ‘Ergonomics’ (29.9 %, n = 660), only 44.6 % (n = 295) said that they were aware about the correct postures/equipment placement and implemented them at the workplace. The commonest reasons for non-implementation were; the lack of proper facilities (34.6 %) and being not convinced of the impact (25.5 %). The mean score for the ten pictorial questions were 5.8 ± 2.3 (range 0–9). The mean score in those with and without CVS were 6.2 ± 1.9 and 5.1 ± 2.7 respectively (p < 0.001).

### Results of the logistic regression analysis

The results of the binary logistic regression analysis in all adults using the dichotomous variable ‘presence of CVS’ as the dependant factor and other independent variables are shown in Table 3. The overall model was statistically significant and the Cox and Snell R-Square and Nagelkerke R Square values were 0.166 and 0.231 respectively. The results indicate that female gender (OR: 1.28), duration of occupation (OR: 1.07), daily computer usage (1.10), pre-existing eye disease (OR: 4.49), not using a VDT filter (OR: 1.02), use of contact lenses (OR: 3.21) and ergonomics practices knowledge (OR: 1.24) all were associated with significantly increased risk of developing CVS (Table 3). Daily computer usage, pre-existing eye disease, using a VDT filter and ergonomics practices knowledge were also associated with CVS in both males and females independently (Table 3). However, duration of occupation and use of contact lenses were not associated in females. In the regression analysis evaluating the factors associated with the severity of CVS, only duration of occupation (OR: 1.04) and presence of pre-existing eye disease (OR: 1.54) were significantly associated with the presence of ‘severe CVS’.

**Table 3:** Results of binary logistic regression of computer vision syndrome in all adults, males and females

Risk factors	Odds ratio (95 % CI)		
	All adults	Male	Female
Female gender	1.28 (1.05–1.57)*		
Age	0.98 (0.97–1.00)	0.95 (0.92–1.01)	1.03 (1.01–1.06)^¥^
Duration of occupation (years)	1.07 (1.04–1.10)^#^	1.12 (1.08–1.17)^#^	NA
Daily computer usage (hours)	1.10 (1.07–1.14)^#^	1.16 (1.11–1.21)^#^	1.06 (1.02–1.10)^¥^
Pre-existing eye disease	4.49 (3.33–6.03)^#^	4.38 (2.96–6.48)^#^	5.20 (3.25–8.34)^#^
Not using a VDT filter	1.02 (1.01–1.03)*	1.01 (1.00–1.02)^¥^	1.02 (1.01–1.03)^#^
Adjusting brightness of screen	0.74 (0.61–1.01)	0.73 (0.55–0.98)	0.67 (0.50–1.01)
Angle of Gaze	1.01 (0.91–1.10)	NA	1.01 (0.99–1.02)
Use of contact lenses	3.21 (1.09–9.47)*	5.25 (1.12–24.5)*	NA
Ergonomic knowledge	1.24 (1.19–1.30)^#^	1.26 (1.19–1.34)^#^	1.23 (1.15–1.31)^#^

*NA* not associated

*VDT* visual display terminal

* p < 0.05

^¥^p < 0.01

^#^p < 0.001

## Discussion

In this first comprehensive national survey on the prevalence of CVS in computer office workers from a South Asian country, the 1 year prevalence of CVS was 67.4 %. Previous studies on CVS from Malaysia (68.1 %) and Nigeria (74.0 %) have demonstrated similar results [13, 17]. In contrast, another study among medical and engineering students in Chennai has found a higher prevalence of CVS (80.3 %) [14], whereas a study among keyboard users in Mauritius has found a lower prevalence of CVS (59.9 %) [18]. The higher prevalence observed in the study from Chennai (80.3 %) is possibly due to the involvement of neck and shoulder pain as a symptom of CVS by the study team, whereas our definition of CVS consisted only of eye/visual symptoms apart from headache. Also in our study only the symptoms which lasted at least 1 week were considered as symptoms of CVS whereas they had no specification on duration of symptoms and therefore included even transient symptoms [14].

The most common symptom reported in the present cohort was headache (45.7 %), followed by dry eyes (31.1 %) and pain in and around the eyes (28.7 %). Megwas and Daguboshim reported that headache (41.8 %), pain (31.6 %) and eye strain (26.7 %) were the most prevalent visual symptoms among VDT users [19]. Headache was the most commonly reported symptom in computer users in several other similar studies [13, 20, 21]. Headaches is often accompanied by other symptoms of CVS, though many patients do not consider it to be a directly vision-related problem [22]. Human eyes need to adjust themselves in order to see objects from different distances, such as by changing the size of pupil, lengthening or shortening the lens to change eye focus, and contracting extra-ocular muscles to coordinate the two eyes. If computer user needs to view computer screen while looking at a paper on the table from time to time, the eyes have to adjust constantly. In addition, the words and images on a computer screen are difficult for the eyes to focus on due to their poor edge resolution. The eyes tend to change the focus to a resting point and then refocus on the screen. For these reasons, constant focusing and refocusing is required. These constant changes take place thousands of times a day when a computer user stares at a computer screen for hours, which then stresses the eye muscles leading to eye fatigue and discomfort causing headaches [23].

According to the results of the binary logistic regression analysis, the most significant risk factor for development of CVS was pre-existing eye disease (OR: 4.49) followed by use of contact lenses (OR: 3.21). Supporting this finding, a study done in Malaysia has revealed that use of correction spectacle/lenses were significantly associated with CVS (OR: 1.91) in multivariate logistic regression analysis, even after adjustment for other confounding variables [17]. Furthermore, university students who were wearing spectacles experienced symptoms of CVS significantly more often than those who were not wearing spectacles [20]. A study by Logaraj et al. also revealed that medical and engineering students wearing corrective lens (spectacle or contact lens) showed a significantly higher risk of developing headache (OR: 1.80) and blurred vision (OR: 2.10) [14]. Possible explanations for the increased risk of CVS among those using correction spectacles/lenses is because the computer tasks are types of near work where letters on the screen are formed by tiny dots called pixels, rather than a solid image, it causes the eyes which already have some corrective problem to work a bit harder to keep the images in focus [17].

Female gender was also significantly associated with the risk of developing CVS (OR: 1.28). Many studies have reported a significant association between female gender and prevalence of CVS [17, 18, 24]. However, when considering individual symptoms, Logaraj et al. reported redness, burning sensation, blurred vision and dry eyes were comparatively more in males than in females [14]. Our study revealed prevalence of red eyes, changes in visualizing colours and excessive tearing were significantly higher among males. Daily computer usage (OR: 1.10) and duration of occupation (OR: 1.07) also significantly predicted the risk of CVS. Evidence from many other studies supports these findings [13, 20, 24]. Rahman and Sanip, in their study reported that spending more than 7 h per day on computer at work was a significant predictor for CVS (OR: 2.01) [17]. Mutti and Zandic reported more pronounced visual symptoms in people spending 6–9 h daily at a computer [25], while Stella et al. observed the same in people using computer more than 8 h daily [26]. However present study did not reveal a significant association of daily computer usage with presence of severe CVS.

Present study demonstrated ergonomics practices knowledge (OR: 1.24) was associated significantly with increased risk of developing CVS. This may be because ergonomics practices knowledge is higher among frequent computer users with long duration of occupation and higher daily computer usage than among infrequent computer users. Therefore prevalence of CVS could be increased among those with higher ergonomics practices knowledge as increased duration of occupation and daily computer usage are significant risk factors for CVS. Although ergonomics practices knowledge is present, lack of implementation of ergonomic practices knowledge at their work place also may be reason for higher prevalence of CVS. Strengthening the fact, present study demonstrated that in those who had heard of the term ‘Ergonomics’, only 44.6 % implemented them at work place. Results from a study by Khan et al. are as follows; as far as the distance from the computer screen was concerned, 42 % respondents were aware, while only 32 % always maintained it. In the same study, although 55 % knew, only 35 % kept the top line of print at their eye level [27].

Lack of a VDT filters significantly predicted (OR: 1.02) the risk of CVS. Use of antiglare filters over VDT screens has been associated with shorter, less frequent and less intense eye complaints in some studies [28]. Significantly lower prevalence of visual complaints in the subjects who used antiglare screen was also observed by Saurabh et al. [29]. However in contrast to this, study by Reddy et al. reported that the use of VDT filters did not help in reducing the symptoms of CVS [20]. According to the binary logistic regression analysis, increasing age was a significant risk factor for CVS only in females. In contrast to this, Rahman and Sanip reported age group of less than 27 years old was a significant predictor for CVS (OR: 2.89). The explanation for this finding as given by them was a significant negative correlation between age of the respondents and duration of computer usage at work (*r* = 0.213, p < 0.001) [17]. In our study population although there was no significant correlation between age and daily computer usage, a significant positive correlation was found between age of respondents and duration of occupation in all adults (*r*: +0.69, p < 0.001) and in females (*r*: +0.70, p < 0.001). This explains higher prevalence of CVS in older age.

Adjusting the brightness of screen and angle of gaze were not significantly associated with CVS in regression analysis. However Stella et al. reported that respondents employing a gaze angle of less than 15° recorded the lowest visual complaints whereas visual complaints were more pronounced with viewing angles of 30–50° [26]. Improper viewing angle has been identified as a factor contributing to CVS in a review article on CVS [30]. It is recommended that the screen should be placed 10–20° below the eye level [31]. Higher viewing angles expose a greater area of conjunctiva and cornea to air and increase the chances of irritant-like symptoms [32]. Although not significant in regression, our study also reported mean angle of gaze to the monitor was significantly higher in those with CVS than in those without CVS. Significantly higher risk of developing visual symptoms was reported among students viewing computer at a distance of less than 20 inches by a previous study [33]. Another study supported this finding by reporting more pronounced visual symptoms when computer is viewed at distance less than 10 inches [26]. However present study revealed there is no significant association between distance from face to monitor and CVS. This may be due to inaccurate measurements reported by respondents. Taking a break was not significantly associated with prevalence of CVS as demonstrated by present data. Reddy et al. reported a similar finding [20]. In contrast to these, several studies reported a significant risk of getting visual symptoms when not taking frequent breaks [14, 17, 33].

The main limitations of this study were that it was a cross-sectional study and it did not include ophthalmic examinations and the symptoms reported were self reported. There are several limitations to our study; the cross sectional design limits the inference of causality and can only demonstrates an association between CVS and identified risk factors. Hence, prospective follow up studies among computer office workers without CVS is required to identify risk factors for CVS during subsequent follow up. Not including neck and shoulder pain as a symptom of CVS was also a limitation. It has been considered as an extra-ocular symptom of CVS in many studies and reviews on CVS [14, 20, 21, 23, 30]. Since the study did not involve examination of their practices while they were actually working on their computers self reported measurements like viewing distance and length of time they work may be less accurate.

## Conclusions

Sri Lankan computer office workers had a high prevalence of Computer Vision Syndrome. Female gender, longer duration of occupation, higher daily computer usage, pre-existing eye disease, not using a VDT filter, use of contact lenses and higher ergonomics practices knowledge all were associated with significantly with the presence of CVS. The factors associated with the severity of CVS were the duration of occupation, lower ergonomics practices knowledge and presence of pre-existing eye disease. However, further prospective follow up studies are required to establish causality for identified risk factors.

## Abbreviations

CVS: computer vision syndrome
NA: not associated
NS: not significant
OR: odds ratio
OSHA: occupational safety and health administration
PPS: probability proportionate to sample size
SPSS: Statistical Package for Social Sciences
VDT: visual display terminal
